# Renal denervation in a patient with a highly tortuous renal artery using a guide extension catheter: a case report

**DOI:** 10.1186/s12872-021-02199-9

**Published:** 2021-08-10

**Authors:** Peijiang Wang, Jindong Wan, Jixin Hou, Sen Liu, Fei Ran

**Affiliations:** Department of Cardiology, Clinical Medical College, The First Affiliated Hospital of Chengdu Medical College, Chengdu, 610500 Sichuan China

**Keywords:** Renal denervation, Tortuous renal artery, Resistant hypertension, Extension catheter, Case report

## Abstract

**Background:**

Catheter-based renal denervation (RDN) has been introduced to treat resistant hypertension. Although the technology of RDN has been largely improved, denervation of tortuous renal arteries remains challenging.

**Case presentation:**

This is a case report of a 49-year-old man with drug resistant hypertension. The patient was selected for RDN after ruling out possible causes of secondary hypertension. Computed tomography angiography showed a highly tortuous left renal artery. An Iberis multielectrode ablation catheter failed to reach the target vessel with a regular guiding catheter. A 5-French extension catheter was introduced into the proximal segment of the main left renal artery to provide extra support force, which enabled successful ablation of the highly tortuous left renal artery. His ambulatory blood pressure was significantly decreased at 1 month follow-up.

**Conclusions:**

It is feasible and effective to use a guide extension catheter for denervation of highly tortuous renal arteries. The present study provides a useful method to ablate tortuous and angled renal arteries and branches.

## Background

Renal sympathetic overactivity contributes to the development and maintenance of resistant hypertension. Catheter-based radiofrequency renal denervation (RDN) has been introduced to treat resistant hypertension for more than a decade. Previously, single-electrode radiofrequency ablation systems achieved inconsistent blood pressure–lowering effects, throwing RDN into debate. The development of multielectrode radiofrequency ablation systems has improved the reproducibility and completeness of RDN, yielding consistent anti-hypertensive effects [1]. However, denervation of tortuous renal arteries remains challenging. It is extremely difficult to obtain an adequate arterial wall contact at all ablation sites in tortuous renal arteries, which often leads to procedure failure [2]. Patients with tortuous renal arteries are usually excluded from clinical trials of RDN [3], and future studies need to focus on optimal deliverability of ablation to tortuous renal arteries [4]. Therefore, there is lack of clinical evidence or recommendations regarding the strategy for ablating tortuous renal arteries [4, 5]. In this report, we present a RDN strategy for a highly tortuous renal artery using a guide extension catheter.

## Case presentation

We present a 49-year-old man with a 5-year history of hypertension. His blood pressure was uncontrolled despite a combination of five different antihypertensive drugs at target doses [6] (valsartan 80 mg, amlodipine 5 mg, bisoprolol 10 mg, hydrochlorothiazide 25 mg, terazosin 5 mg) with optimal patient compliance to therapy. At admission, the average ambulatory blood pressure was 158/100 mmHg with a non-dipper night pattern. The patients had a normal BMI and renal function. He had left ventricular hypertrophy with a mild left ventricular diastolic dysfunction. The recommended work-up has been done to rule out possible causes of secondary hypertension.

**Fig. 1:**
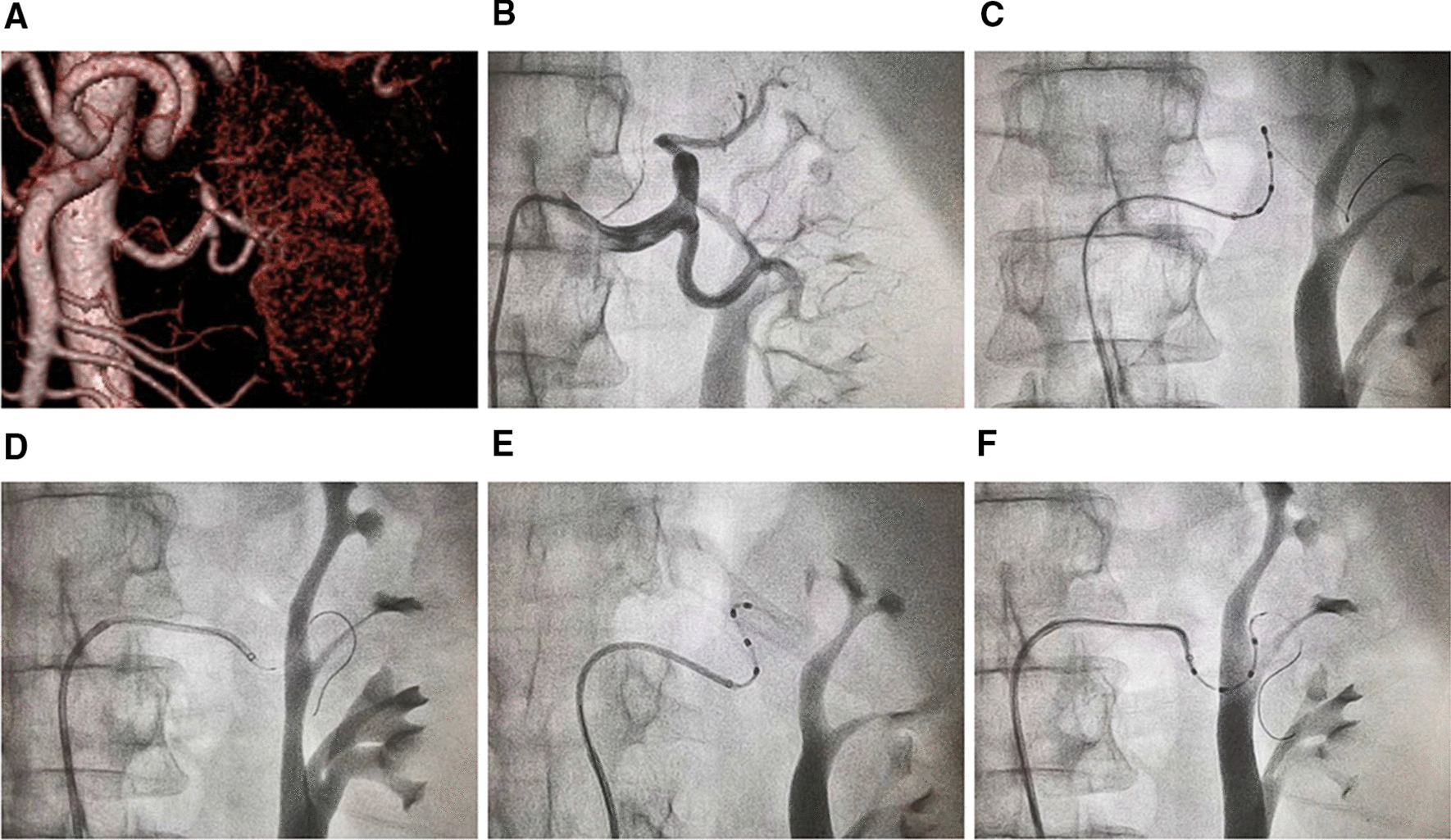
Angiography and denervation of the left renal artery. **a** Three-dimensional reconstruction of computed tomography angiography showing the highly tortuous left renal artery. **b** Angiography of the left renal artery. **c** Attempted ablation using an Iberis ablation catheter with a regular guiding catheter alone. **d** Insertion of a guide extension catheter. **e** Denervation of the upper branch of the left renal artery using a guide extension catheter. **f** Denervation of the lower branch of the left renal artery using a guide extension catheter

**Fig. 2:**
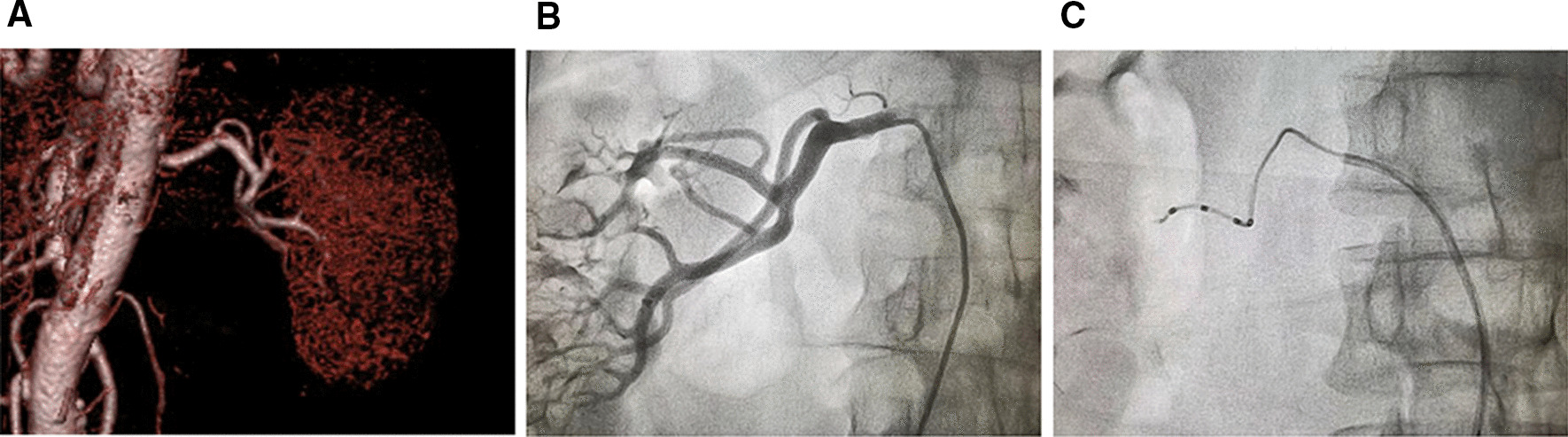
Angiography and denervation of the right renal artery. **a** Three-dimensional reconstruction of computed tomography angiography showing the right renal artery. **b** Angiography of the right renal artery. **c** Denervation of the right renal artery using an Iberis ablation catheter with a regular guiding catheter

Computed tomography angiography (CTA) showed a highly tortuous left renal artery and a relatively normal right renal artery (Figs. 1a and 2a). Given the diagnosis and evaluation of the resistant.

hypertension, the patient was eligible and selected for percutaneous RDN. After local anesthesia with lidocaine, a 6-French sheath was placed into the right femoral artery. Renal artery angiography confirmed the high tortuosity of the left renal artery with no stenosis in either left or right renal arteries (Figs. 1b and 2b). Along a guidewire, an Iberis ablation catheter (Multielectrode Renal Denervation System, AngioCare Medical Technology. Shanghai, China [7]) was inserted into the left renal artery. Due to lack of sufficient support force, the ablation catheter failed to reach the target vessel for ablation after several attempts (Fig. 1c). A 5-French GuideZilla extension catheter (Boston Scientific, Marlborough, MA) introduced into the proximal segment of the main left renal artery to provide extra support force (Fig. 1d). After that, successful ablation was achieved in both upper (Fig. 1e) and lower (Fig. 1f) branches of the left renal artery. Ablation of the right renal artery was successfully performed without using an extension catheter (Fig. 2c). A total of six ablation sites were performed in a spiral fashion in each renal artery. Post-ablation angiography of bilateral renal arteries showed no vasospasm, stenosis, or dissection. No vascular or renal complications occurred during hospitalization.

At 1 month follow-up, the average ambulatory blood pressure was 140/90 mmHg with a significant reduction of 18/10 mmHg, and his renal function was normal. During the 6 months after the procedure, the antihypertensive drugs were decreased from five to three drugs (valsartan 80 mg, amlodipine 5 mg, hydrochlorothiazide 25 mg).

## Discussion and conclusions

The crucial role of renal sympathetic overactivity in essential hypertension was appreciated decades ago. Overactivation of renal sympathetic nerves raises blood pressure through vasoconstriction and volume/sodium retention mechanisms. Since then, surgical sympathectomy had been attempted to treat patients with hypertension but had been abandoned due to severe side effects [8]. Selective renal sympathetic denervation achieved by catheter-based percutaneous RDN was introduced to treat resistant hypertension in 2009 [9]. This strategy brought new hope of sympatholytic therapy for treating drug-resistant or refractory hypertension. Anatomy of both renal arteries and nerves is always a challenge for this new technology [10]. For example, patients with small, multiple, or highly tortuous renal arteries are usually excluded from clinical trials of RDN [3]. Ablation of small renal arteries has been largely improved by using newly-designed smaller diameter catheters [10], while denervation of highly tortuous renal arteries remains challenging.

Since clinical trials usually exclude patients with highly tortuous renal arteries, there is lack of evidence on how to denervate this type of vessels. According to 2019 Taiwan Consensus Statement on Renal Denervation, a guidewire with strong support is recommended for cases with tortuous and angulated renal arteries [11]. The present study demonstrated successful ablation of a highly tortuous renal artery using a guide extension catheter. A guide extension catheter would provide better support than using a regular guiding catheter. This study provides a novel and feasible strategy for denervation of highly tortuous renal arteries.

In conclusion, it is feasible and effective to use a guide extension catheter for denervation of highly tortuous renal arteries.

## Data Availability

Data sharing is not applicable to this article as no datasets were generated or analyzed during the current study.

## Abbreviations

RDN: Renal denervation
CTA: Computed tomography angiography
